# Crosstalk between Tumor Cells and Immune System Leads to Epithelial-Mesenchymal Transition Induction and Breast Cancer Progression

**DOI:** 10.29252/ibj.25.1.1

**Published:** 2020-07-29

**Authors:** Raheleh Moradpoor, Mona Salimi

**Affiliations:** 1 Department of Basic Sciences, School of Allied Medical Sciences, Shahid Beheshti University of Medical Sciences, Tehran, Iran;; 2 Department of Physiology and Pharmacology, Pasteur Institute of Iran, Tehran, Iran

**Keywords:** Chemokines, Cytokines, Epithelial-mesenchymal transition, Tumor microenvironment

## Abstract

Herein, we review the current findings of how a variety of accessory cells could participate in shaping the TME and supporting the mechanisms by which cancer cells undertake the EMT. EMT, a complex of phenotypic changes, promotes cancer cell invasion and creates resistance to chemotherapies. Among the accessory cells present in the EMT, immune cells (both native and adaptive) can reciprocally influence the tumor cells features, promote EMT and negatively regulate the anticancer immune response. In this review, we look over the role of EMT in crosstalk between tumor cells and the immune system, with specific emphasis on breast tumors. Finally, we suggest that understanding the role of immune cells in cancer progression could create new opportunities for diagnostic and therapeutic interventions in cancer combination therapy.

## INTRODUCTION

The TME consists of tumor cells plus a variety of accessory stroma cell types that may have a selective advantage in tumor survival and metastasis as a result of crosstalk between tumor and stroma cells^[^^1^^]^. Infiltrating immune cells, vascular endothelial cells, and cancer-associated fibroblasts are examples of the cells present in the TME and serve unique roles in allowing cancer cells to acquire phenotypes in favor or to the detriment of tumor progression^[^^1^^,^^2^^]^, the latter of which is the focus of this report. Comprehensive understanding of the TME of breast cancer has revealed strong evidence to propose that TME and the associated molecules contribute to the development of tumor growth and metastasis. 

The critical elements of TME in breast cancer may help us to discover the new biomarkers, including immunological and immunosuppressive markers with a function in tumor progression^[^^3^^]^. In this context, the role of immune cells in EMT have been well studied^[^^4^^,^^5^^]^. 

EMT process is a transition from non-motile to motile cells in which tumor cells lose cellular polarity due to certain molecular changes, including the loss of E-cadherin and occluding as well as the gain of vimentin, fibronectin, and N-cadherin^[^^6^^,^^7^^]^. Indeed, EMT is the main cause of heterogeneity of carcinoma cells, resulting in the alteration of their biological functions and phenotypic characteristics^[^^8^^]^. It is worth mentioning that immune editing may cause the tumor cells to lose their capability of expressing tumor antigens, consequently leading to a poor recognition of the tumor cells by the immune cells, and a more enhanced EMT progression^[^^9^^]^. In other words, the immune system creates an immunosuppressive TME, permitting tumor cells to evade the immune recognition and destruction^[^^9^^,^^10^^]^. 

Knowing that tumor stroma cells can either enhance or inhibit tumor cell progression, a great deal of interest has been taken toward elucidating the underlying mechanism behind immune activation or immune tolerance. In the current review, we focus on the immunosuppressive role of the stroma cells in favor of tumor progression. In order to define the role of immune system in EMT process, it is worthy to describe different types of immune cells in TME. In this regard, recent studies have highlighted that EMT is associated with the presence of innate immune cells, including TAMs polarized to M2, MDSCs, TANs and NK cells along with adaptive immune cells compromising two broad types of T lymphocyte (CD4^+^ and CD8^+^ T cells) and Tregs^[^^11^^-^^15^^]^. 

**Key immune cell types in TME**

One of the cell populations of the innate immune system influencing the TME is macrophages. They originate from the circulating monocytes that are recruited to TME, mostly by chemokines such as MCP-1/CCL2 and CSF-1 derived from tumor cells or mesenchymal stem cells, which were later polarized and denoted as TAMs^[^^7^^,^^16^^]^. Besides chemokines, TAMs can be recruited by hypoxia condition presents in tumors, which subsequently increase the hypoxia-inducible factor-α production and pro-angiogenesis factors like VEGF, basic fibroblast growth factor, TNF-α, and CXCL12, assisting in remodeling the extracellular matrix and causing angiogenesis promotion^[^^17^^-^^19^^]^. It has been reported that isolated TAMs from breast cancer tissues produce high amounts of pro-tumor cytokines, including CCL18, CCL17, CCL22, and IL-10 and reveal a CD206^high^/human leukocyte antigen-DR^low^ phenotype relating to the TME immunosuppressive phenotype^[^^20^^]^. Interestingly, TAMs can acquire different functional phenotypes depending on TME signals; they are capable of having either the classical anti-tumor M1 type in response to IFN-γ or lipopolysaccharide^[^^21^^]^ or the alternative pro-tumor M2 type by IL-4, IL-10, IL-13, TGF-β and lactic acid^[20]^. Behavior of the tumor cells varies according to the type of TAMs. In this context, M1 TAMs are highly phagocytic and influence the Th1 response through the secretion of the inflammatory factors like IL-1, IL-6, IL-12, and TNF-α, reactive oxygen species, and inducible nitric oxide synthase^[^^21^^,^^22^^]^. Contrary to M1, M2 TAMs participate in Th2 response and cancer progression and affect anticancer therapies via the secretion of the immunosuppressive cytokine such as TGF-β and mitogenic growth factors, including PDGF and epidermal growth factor^[^^23^^,^^24^^]^. The effect of TAMs is proportionate to the level of cytokines in TME, i.e. the low level of TNF-α causes inflammation and cell survival and upregulates negative regulators of apoptosis; however, the high level of TNF-α promotes the apoptosis and cell death. 

Beside cytokines, TAMs are known to enhance the expression of COX2, which correlates with the secretion of IL-6, PGE2, and MMP9 and promotes EMT process in cancer cells, including breast cancer, by activating the Akt pathway and stabilizing the EMT-promoting transcription factors like SNAIL^[^^22^^,^^24^^]^. Furthermore, the positive feedback loop between GM-CSF secreted from breast cancer cells and CCL18 from M2-TAMs facilitates EMT process, induces tumor progression and reduces the patient’s survival in breast cancer^[^^20^^]^. Moreover, GM-CSF triggers the function of the transcription factors such as STAT5, NF-B as well as ERK signaling in TAMs to increase their recruitment and enhance their polarization to M2 type and finally cause TGF-1 expression^[^^25^^]^. It is important to point out that due to a strong correlation between ferritin light chain released from M2-TAMs and aggressive phenotype of breast tumors, these tumors have to be more intensively followed-up^[^^26^^]^. Interestingly, TAMs constitute a major component (5-40%) of the tumor mass in breast cancers, which are found fourfold higher than normal mass in the early benign proliferative regions, while they increase to twentyfold in the invasive front of the tumors where EMT is usually initiated. This observation could be suggested as a diagnostic tool to distinguish the metastatic from non-metastatic region^[^^22^^,^^27^^]^. In line with it, the metastatic tumor microenvironment is characterized by staining three parts, including migrative cancer cells, TAMs, and endothelial cells. In breast cancer, in particular, tumor microenvironment of metastasis facilitates metastasis, and due to this reason, it is proposed as a promising target in clinical application and drug development^[^^24^^]^. In addition, vessel-associated macrophages assist the intravasation of cancer cells into vasculature through the secretion of epidermal growth factor, which it is crucial for EMT induction^[^^24^^]^. Overall, it can be suggested that M2 TAMs promote the cancer cells toward EMT and might have a chance in remodeling of TME^[^^20^^]^. More notable is that chemotherapy combining with M2 TAM deletion would provide an encouraging approach in cancer therapy^[^^26^^]^. 

***Tumor-infiltrating lymphocytes***

EMT process is associated with a decrease in the number of CD4^+^ and CD8^+^ T cells in TME, which is likely related to the expression of  immunosuppressive cytokines such as IL-10 and TGF-β^[^^28^^]^ as well as to the inhibitory immune checkpoint molecules, including PD-L1, PD-L2, cytotoxic T-lymphocyte antigen-4, T-cell immunoglobulin and mucin domain-containing-3, B7-H3, CD73, and CD47^[^^28^^-^^30^^]^. Breast cancer progression could also be affected by dysregulating different T-cell subsets in TME. In this regard, the presence of tumor-infiltrating lymphocytes in TNBC and human epidermal growth factor receptor 2^+^ breast tumor subtypes is associated with a good prognosis as well as favorable chemotherapy response^[^^31^^]^. On the contrary,  the high level of TGF-β, as an effector cytokine influencing the differentiation of CD4^+^, promotes the differentiation of CD4^+^FOXP3^+^ Tregs and inhibits the function of Th1 cells^[^^32^^]^. Importantly, Tregs have a decisive role in the suppression of TME^[^^33^^]^. It is significant to note that human epidermal growth factor receptor 2-positive breast cancer individuals have a higher level of Tregs compared with the healthy ones^[^^10^^]^. EMT mediated the expression of immunosuppressive molecules like indoleamine 2,3-dioxygenase in TAMs and upregulation of an extracellular matrix protein such as SPARC in BBCs, which promotes the infiltration of Tregs, mast cells, and MDSCs into TME^[^^33^^-^^35^^]^. Nonetheless, SPARC is a protein with dual functions, of which its immune-suppressive activity is the subject of interest, considering its relatedness to EMT^[^^36^^]^. 

Furthermore, immune checkpoint molecules can regulate EMT process. It was revealed that the expression of PD-L1 on breast tumors is connected with resistance to CD8^+^-mediated cell killing^[^^37^^]^. On the other side, PD-L1 expression in the aggressive tumor cells induces PD-1 on T cells, which consequently dampens cytotoxic T-lymphocyte attack, resulting in the escape of tumor cells from recognition by the immune system^[^^38^^]^. Although PD-L1 expression is significantly higher in the invasive than non-invasive breast cancers, it is promising due to favorable outcomes of recent monoclonal antibodies against PD-1 or PD-L1 in cancer immunotherapy^[^^39^^]^. Among the immune checkpoints, tumor-derived CD73 in human breast cancers was also found to significantly suppress CTL and NK responses^[^^40^^]^. In this context, CD4^+^ Foxp3^+^ Tregs are a key source of host CD73 in TME, which is related to poor prognosis and chemoresistance in the TNBC and contributes to EMT-mediated trastuzumab resistance and TGF-β–mediated tumor immune escape^[^^40^^]^. 

A T-cell subpopulation that is well-known for the anti-tumor function is NKT cells, which act as a bridge between the innate and adaptive immune system^[^^41^^,^^42^^]^. These types of T cells in collaboration with NK-cells and Th1 cytokines demonstrate a strong anti-tumor immunity. Interestingly, Tregs can suppress differentiation of NKT cells leading to reduction in the number of NKT cells in the advanced cancer patients. Meanwhile, MDSCs could also prevent the anti-tumor response of NKT cells via TGF-β production^[^^10^^,^^43^^]^. Indeed, if the immunosuppressive feature of the microenvironment overcomes due to the presence of both immunosuppressive factors and cells, it would lead to tumor survival and eventual cancer progression. 

***NK cells***

NK cells are innate lymphoid cells known for their immune surveillance function against cancer cells, which is dependent on the balance between NK cell-activating and -inhibiting ligands expressing in tumor cells^[^^44^^,^^45^^]^. Indeed, NK cells are heterogeneous and characterized by two common phenotypes: CD56^bright^CD16^dim/neg ^(CD56^bright^) and the CD56^dim^/CD16^bright^ (CD56^dim^)^[^^46^^,^^47^^]^. The CD56^bright^ NK cells mostly enhance IFN-γ production and represent a strong cytotoxic function^[^^48^^]^. Interestingly, breast tumors recruit the CD^56bright^ NK cells into the TME through releasing a high level of CCL19 and a low level of CXCL12, highlighting the role of NK cells in cancer patient’s survival^[^^49^^]^. EMT process reduces E-cadherin and induces cell adhesion molecule 1 expression in the tumor cells, leading to the enhancement of NK cell cytotoxicity susceptibility^[^^46^^]^. In this context, it has been shown that the upregulation of cell adhesion molecule 1, as an NK cell-activating ligand, is associated with the patient’s survival in breast cancer individuals and results in the metastasis reduction^[^^44^^,^^46^^]^. It is important to note that the high cytotoxic capacity of NK cells belongs to the ones present in the blood circulation and lymph nodes, which can eliminate the disseminated metastatic cancer cells within the first 24 hours^[^^50^^]^. It has been suggested that breast tumors, independent of the subtype, secrete a panel of factors that modify NK cell functions causing tumor cells to escape from anti-tumor immunity function of NK cells^[49]^. It has also been reported that EMT inducer factors such as TGF-β1, PGE2, indoleamine 2,3-dioxygenase, sMICA, and LGALS3 are produced by a variety of immune suppressor cells like tumor-associated fibroblasts, Tregs, MDSCs and even tumor cells attenuated NK cell-mediated cytotoxicity^[^^51^^,^^52^^]^. Another important event occurs in TME is the expression of the immune-checkpoints such as PD-L1, T-cell immunoglobulin and mucin domain-containing-3, TIGIT, and SIGIRR by NK cells similar to that happens in tumors, which results in tumor adaptive resistance to NK cells immune surveillance^[^^53^^]^. Importantly, using the specific monoclonal antibodies for blocking these checkpoints can improve the NK-mediated cytotoxicity and inhibit metastasis dissemination^[^^53^^]^.

***TANs***

Neutrophils are abundant myeloid-derived circulating cells, although they can migrate to a number of tissues^[^^54^^]^. In this context, accumulating evidence has demonstrated that neutrophils constitute a significant part of the TME *as* TANs *with both pro- and anti-tumorigenic properties*^[^^55^^]^. TANs are recruited into TME through releasing the cytokines consisting of IL-8, G-CSF, and IL-17 by tumor cells^[^^56^^]^. TANs frequently represent two phenotypes: anti-tumorigenic (N1) or pro-tumorigenic (N2) phenotype^[^^56^^]^. N1 TANs upregulate OSM upon interaction with BBCs, leading to an inhibitory effect on tumor cell proliferation. In contrast, some studies have revealed that neutrophils upregulate the OSM upon GM-CSF produced by BBCs, which in turn enhances VEGF production and promotes tumor growth and metastasis^[^^57^^]^. In another study, *cathepsin G has been reported as* a neutrophil*-derived* serine *protease*, which enhances migration and invasion potential of breast tumor cells^[^^49^^]^. N2 TANs can educate the other immune cells toward the pro-tumor type in the TME and further stimulate angiogenesis, leading to the poor prognosis of patients. In other words, neutrophils secrete TGF-β and OSM within the TME and drive the macrophages differentiation toward M2 type^[^^58^^]^. Remarkably, neutrophils interplay with T cells in the TME, i.e. T cells enhance the G-CSF expression, which further leads to neutrophil expansion and modifies the neutrophil phenotype^[^^59^^]^. Then the altered neutrophils release inducible nitric oxide synthase, which subsequently reduces the cytotoxicity of CD8 T cells in the TME and enhances breast cancer progression^[^^59^^]^. A strong correlation presents between N2 TAN in the TME and breast cancer subtypes, as TANs are predominantly observed in TNBC subtype of breast cancer^[^^3^^]^. Consistently, a high expression level of TGF-β was observed in TNBC contributing to the neutrophil chemotaxis; however, TGF-β may also induce a pro-tumorigenic N2 TAN phenotype^[^^3^^]^. Moreover, tumor cells could activate neutrophils in a cell-by-cell contact manner causing the expression of hyaluronan from tumor cells that effectively promotes tumor cell migration^[^^60^^]^. In addition, some studies have pointed out the importance of TANs in cancer progression in the late-stage of tumors wherein chronic inflammation could be developed. Inversely, TANs in the early-stage of tumors may exert anti-tumor properties^[^^49^^]^.

*MDSCs*

MDSCs often arise as a result of cytokines such as IL‐1β, IL‐6, and IL‐8 in TME^[^^61^^]^. Migration of MDSCs to breast tumors is regulated by kruppel-like factor-4 transcription factor through CXCL5/CXCR2 axis, leading to EMT process^[^^62^^]^. MDSCs are heterogeneous immature myeloid cells that develop in spleen, peripheral blood, or tumor tissues with potent immune suppressive activities in TME and contribute to tumor growth and resistance to various chemotherapies^[^^63^^]^. MDSCs, mostly consist of two subsets: the monocytic (M)-MDSC (CD11b^+^ Ly6G^−^ Ly6C^hi^) and the polymorphonuclear-MDSC (CD11b^+ ^Ly6G^+^ Ly6C^lo^). However, it has been reported that CD45^+^Ly6G^mi^Ly6C^lo^CD11b^+^ is the dominant phenotype recruiting the TME of aggressive breast cancer. SPARC and CXCR2 are two factors expressed in MDSCs and required for the acquisition of MDSC suppressive phenotype^[^^62^^,^^64^^,^^65^^]^. MDSC differentiation is facilitated by the tumor-derived cytokines, including G-CSF, GM-CSF, VEGF, and chemokines such as CCL2 and CXCL12^[^^62^^,^^66^^-^^69^^]^. Moreover, Thrombospondin 1 expression in the surface of MDSC-derived exosomes also causes MDSCs chemotaxis and migration^[^^64^^]^. 

Of note, in the crosstalk between MDSCs and T cells in TME, activated T cells stimulate STAT3 phosphorylation on MDSCs through IL-10, resulting in the expression of immune checkpoint B7-H1^[^^70^^]^. On the other hand, the expression of B7-H1 ligands and MHC class II on MDSCs causes the upregulation of two inhibitory molecules, PD-1 and LAG-3, on T cells, which is associated with T cell dysfunction and immunosuppressive conditions^[^^70^^,^^71^^]^. Additionally, MDSCs play a significant role in FoxP3^+^ Tregs development as well as the expression of immunosuppressive factors like IL-10 and COX2, which suppress T cells immune response^[^^65^^,^^72^^]^. The literature survey has demonstrated that the targeted depletion of MDSCs in various cancers increases the adaptive immunity and remodels the TME^[^^73^^]^. 

In conclusion, it would seem that broad determining role of immune cells recruited to the tumor site through cytokines and chemokines influence cancer progression, which in turn expand the new opportunities for therapeutic interventions in cancer combination therapy. By using agents to target simultaneously cancer and stroma cells, the survival outcomes and quality of life would be positively altered. Moreover, the stroma compartments consist of potential and specific tumor biomarkers that would be valuable to assess the metastatic stage of cancer. 

## CONFLICT OF INTEREST. 

None declared.
